# Comparison of the Changes in Quality-of-Life of Cats with Hyperthyroidism Treated with Radioiodine or Antithyroid Drugs—A Preliminary Study †

**DOI:** 10.3390/vetsci12060572

**Published:** 2025-06-11

**Authors:** Fabienne Blunschi, Sofie Muthmann, Natali Bauer, Katarina Hazuchova

**Affiliations:** Clinic for Small Animals (Internal Medicine, Clinical Pathology and Clinical Pathophysiology), Justus-Liebig-University,35392 Giessen, Germany; fabienne.blunschi@vetmed.uni-giessen.de (F.B.); sofie.muthmann@vetmed.uni-giessen.de (S.M.); natali.bauer@vetmed.uni-giessen.de (N.B.)

**Keywords:** feline, questionnaire, treatment, welfare, endocrinology

## Abstract

A previous cross-sectional study found impaired health-related quality of life (HRQoL) in hyperthyroid cats, but the longitudinal impact of treatment—especially differences between radioiodine (RAIT) and antithyroid drug (ATD) approaches—remains unclear. This study aimed to evaluate changes in HRQoL in recently diagnosed (<6 months) hyperthyroid cats that underwent RAIT or received ATD. HRQoL was assessed using the validated HyperthyroidismQoL-cat questionnaire at baseline and after 1, 3, and 6 months, alongside thyroid status (eu-/hypo-/hyperthyroid) evaluation. Mixed-effects model analysed the influence of treatment type, timepoint, and thyroid status on log(HRQoL). HRQoL differences between groups at baseline and comparison to a non-hyperthyroid control group (n = 322) from a previous study at month 6 were examined using Mann–Whitney U tests. Data are presented as median (range), with significance set at *p* < 0.05. Thirty-eight client-owned hyperthyroid cats (15 ATD, 23 RAIT) were included. HRQoL scores at baseline did not differ between groups (RAIT: 103.5 [27–211], ATD: 73 [22–260], *p* = 0.22). HRQoL significantly improved over time (*p* < 0.001) but was not affected by treatment type (*p* = 0.20) or thyroid status (*p* = 0.40). Despite improvement, HRQoL remained lower than in non-hyperthyroid controls (hyperthyroid: 42.5 [3–161.5], non-hyperthyroid: 27 [0–249], *p* = 0.007). This study highlights the overall positive impact of treatment on HRQoL, but due to the lack of randomisation and heterogeneity of subjects and treatment, conclusion have to be considered preliminary.

## Simple Summary

Cats with hyperthyroidism have reduced quality-of-life, but how this is affected by different treatment forms over time is unknown. This study examined how health-related quality-of-life changes in hyperthyroid cats receiving either curative radioiodine therapy or palliative antithyroid medication. Quality of life was evaluated using a validated questionnaire before radioiodine treatment or within six months of starting antithyroid drugs and re-assessed at follow-up one, three, and six months later, alongside thyroid hormone concentrations. The study revealed that cats’ quality-of-life improved over time, regardless of the treatment type or normalisation of thyroid hormone concentrations. However, even after treatment, quality-of-life remained lower than that of cats without hyperthyroidism. This study underscores the need for ongoing monitoring and care to ensure the best possible quality-of-life for affected cats.

## 1. Introduction

Hyperthyroidism is the most common endocrine disease in middle-aged to older cats [1]. Being a multi systemic disease caused by an overproduction of thyroid hormones, affected cats show a wide range of clinical signs including weight loss despite an increased appetite, hyperactivity, vomiting, increased thirst and urination, diarrhoea, susceptibility to respiratory distress and changes in coat condition [2]. A previous study that developed and validated a questionnaire to assess health-related quality-of-life (HRQoL) in cats with hyperthyroidism, identified a negative impact of hyperthyroidism and its treatment on the quality-of-life (QoL), with hyperthyroid cats having a poorer HRQoL compared to cats without any known disease [3]. Surprisingly, treatment modality did not have a significant effect on HRQoL scores [3], even if one would expect cats treated with curative treatment options (i.e., radioiodine treatment [RAIT] and thyroidectomy) to have better QoL than those treated palliatively (i.e., antithyroid drugs [ATD] and low iodine diet). However, this was a cross-sectional study, with treatment outcome (eu- vs. hypothyroidism vs. persistent hyperthyroidism) not assessed at the time of HRQoL-questionnaire completion and not accounted for in the analysis. Given the cross-sectional study design, whether any beneficial effect of one treatment modality over the other exists longitudinally, over a longer period of time, could not be assessed. Indeed, in insulin-treated diabetic cats, the beneficial effect of home blood glucose monitoring on HRQoL was detected in longitudinal fashion [4]. Although no difference in HRQoL between diabetic cats monitored at home and cats monitored at veterinary practices was detected at any timepoint of the study (i.e., cross-sectionally), the HRQoL improved over time in home-monitored cats but not in those monitored at the veterinarian only. Therefore, longitudinal assessment of QoL in hyperthyroid cats seems warranted to fully explore any effect different treatment modalities might have on HRQoL.

Interestingly, several studies in people with hyperthyroidism also reported no difference in QoL depending on the treatment modality [5,6], similar to the findings of the single feline study [3], but one study found that people treated with RAIT had worse QoL in comparison to those undergoing surgery or receiving ATD [7]. Overall, current research in human medicine indicates that the QoL in humans with hyperthyroidism might remain reduced for an extended period of time, often even after restoration of euthyroidism [6,8]. As mentioned above, longitudinal assessment of HRQoL in hyperthyroid cats under treatment has, to the authors’ knowledge, not been reported in veterinary medicine.

The main aim of this study was to compare the changes in HRQoL in cats with hyperthyroidism treated with either RAIT or ATD over a six-month period and assess the effect of the treatment modality on the HRQoL. A secondary aim was to evaluate the effect of the disease control status (hyper-, eu- or hypothyroid) and clinically relevant azotaemia (International Renal Interest Society [IRIS] stage 3 or 4 [9]) on the HRQoL. A third aim was to assess whether HRQoL scores at the final six months re-evaluation differed from a control non-hyperthyroid group from a previous study [3].

## 2. Materials and Methods

### 2.1. Study Design, Inclusion and Exclusion Criteria

This was a prospective study comparing changes in HRQoL assessed using a validated questionnaire (HyperthyroidismQoL-cat) [3] over a period of six months between cats treated with ATD or RAIT. At the end of the study, HRQoL of all included hyperthyroid cats, regardless of treatment modality, was also compared to a non-hyperthyroid control group from a previously published study [3] (see Supplementary Materials for detailed characterisation of the control group). All owners gave written consent with study participation. Ethical approval was not required by the local ethical committee because all diagnostic tests were performed as a part of routine management of cats with hyperthyroidism.

Cats diagnosed with hyperthyroidism and not yet treated or treated with ATD for less than six months were recruited for the study between March 2022 and December 2022. There were no other inclusion criteria upon enrolment, but cats could be later excluded from the analysis if less than two HRQoL questionnaires were completed by the owner, e.g., because the cat died, or the owner decided to withdraw their consent with study participation. Furthermore, if an owner of an ATD-treated cat decided to pursue RAIT, only data up to the timepoint of RAIT were included in the analysis.

The diagnosis of hyperthyroidism was made based on appropriate clinical signs (e.g., weight loss, hyperactivity, vomiting, changes in coat quality) in combination with total thyroxine (TT4) levels increased above the respective laboratory reference range (RR) [10]. In cats undergoing RAIT, technetium-99m thyroid scintigraphy was additionally used to confirm hyperthyroidism before RAIT.

### 2.2. Recruitment and Assignment of Treatment Groups

Cats were grouped according to owner’s choice of treatment. Cats receiving antithyroid drugs (tablets, liquid formulation/syrup or transdermal ointment) were assigned to the ATD-group while cats scheduled for radioiodine therapy were included in the RAIT-group. Cats in the ATD-group were recruited through requests sent to primary care veterinarians by email and via social media. Cats in the RAIT-group were recruited by offering study participation to owners of hyperthyroid cats prior to their scheduled RAIT appointment at the authors’ institution. RAIT-group cats were all treated at the MASKED FOR REVISION with an individually calculated dose of radioactive iodine, based on percent thyroid uptake of technetium (TcTU), with cats with TcTU < 4% receiving a dose of 1–3 mCi (37–111 MBq), and cats with TcTU > 4% receiving 4–6 mCi (148–222 MBq) [11]. Cats with suspected carcinoma were treated with 10–30 mCi (370–1110 MBq) radioiodine. Antithyroid drugs had to be discontinued for one week prior to the RAIT. Cats in the ADT-group were continued to be managed primarily by their primary care veterinarians, with dose adjustments based on the attending clinicians’ discretion. There was no interference with medical decisions by the authors of this study. Participation in the study was entirely voluntary, but owners of RAIT-treated cats were offered a discount on the treatment. There was no incentive for participation for owners of ATD-treated cats.

### 2.3. Assessment of the Quality-of-Life

Owners were asked to complete a previously validated questionnaire (HyperthyroidismQoL-cat) [3] to assess the HRQoL at four timepoints (specified below). The questionnaire was used in German or English language, depending on the preferred language of the owner. The survey was made available to the participants using an online survey programme (LimeSurvey GmbH, Hamburg, Germany) and could only be accessed through a link that was sent to the participants via email at the respective timepoints. The links expired after 30 days.

The HyperthyroidismQoL-cat produces a score between 0 and 382, zero being the best possible HRQoL and 382 the worst. The questions are grouped into four domains (“owner factors”, “dietary, gastrointestinal and urinary signs”, “appearance”, and “activity and behaviour”). An additional question of the tool asks owners to rate their overall impression of their cats QoL with five possible answers from very poor to very good [3].

The first time the questionnaire was completed was at study enrolment, in the RAIT-group this was within one week of the RAIT appointment. The follow-up assessment timepoints were one month, three months and six months after the first questionnaire. The study timepoints were chosen based on the standard recommended re-examination timepoints after the RAIT at the authors’ institution. The questionnaires were identical at all four timepoints and completed by the same owner each time. Owners were not informed about the results of the previous surveys before the study was completed.

### 2.4. Blood Tests and Follow-Up

Measurement of TT4 concentration was required in all cats to assure correct diagnosis of hyperthyroidism, but in ATD-group cats this was not necessarily directly prior to study enrolment.

For the RAIT-group, haematology, biochemistry, TT4 and thyroid stimulating hormone (TSH) measurement were performed as a part of routine work-up prior to RAIT (study timepoint “enrolment”), and biochemistry, TT4 and, where available, TSH were re-assessed at one, three and six months after the RAIT as a part of routine follow-up of RAI-treated cats at our hospital (and also in agreement with other studies) [12,13], corresponding with the scheduled assessments of HRQoL. The follow-up re-examinations took place at the primary care veterinarians, but samples for TT4, TSH and creatinine measurement were sent by post to the Clinic for Small Animals, Justus Liebig University Giessen. Therefore, blood tests of the RAI-treated cats were at all timepoints performed either at the Central Laboratory of the Justus Liebig University Giessen (haematology, biochemistry incl. creatinine) or at an external laboratory (Biocontrol, Ingelheim, Germany; measurement of TT4 and TSH) and measured with chemiluminescent immunoassay. Depending on the results, the owners of RAI-treated cats were advised on treatment if needed (e.g., need for levothyroxine (LT4) replacement, see Section 2.5).

In the ATD-group, re-examinations at 1, 3, and 6 months were recommended. Cats remained under the care of their primary veterinarians, limiting the ability to ensure consistent follow-up. Quality-of-life assessment was performed at the timepoints described above for the RAIT-group, but laboratory results were only included in the statistical analysis if testing was performed within one month prior to or after the completion of the HRQoL-questionnaire at the respective study timepoint. The blood tests in the ATD-group cats were either performed in-house by the primary care veterinarian or sent to an external laboratory, but there was no requirement for using a specific laboratory; accordingly, there was no standardised method for measuring TT4.

### 2.5. Thyroid Status and Azotaemia

Because TT4, TSH and creatinine were measured in several different laboratories, the absolute values of those analytes were not used in the statistical analysis. Instead, thyroid status (eu-, hypo- or hyperthyroidism) and presence of azotaemia were recorded for every cat and every study timepoint (enrolment, month-1, month-3, month-6) with available test results. Azotaemia was defined as creatinine > 140 μmol/L irrespective of the RR of the respective laboratory based on the International Renal Interest Society Guidelines [9], as previously reported [11,14].

In cats of the ATD-group, TSH measurements were not performed by the primary care veterinarians in any case and thyroid status was classified as eu-, hypo- or hyperthyroid based on the TT4 concentration alone in relation to the RR of the respective laboratory. This approach was also used in the RAIT-group on those occasions where TSH measurement was not performed (lacking sufficient blood sample, 11 re-examination timepoints in eight cats). In the majority of re-examinations of cats of the RAIT-group, TSH measurement was available (87% of all re-examinations) and the combination of TT4 and TSH was used to assess thyroid status (specifically to differentiate between eu- and hypothyroidism). Cats with TT4 > RR of the laboratory (TT4 > 51.5 nmol/L) were classified as hyperthyroid. Cats with TT4 and TSH within RR (TT4: 12.9–51.5 nmol/L; TSH < 0.44 µU/mL) were classified as euthyroid, and cats with TT4 below or within lower RR (TT4 < 32.2 nmol/L) with concurrently increased TSH (TSH > 0.44 µU/mL) were classified as hypothyroid [11]. In cats with hypothyroidism and new onset of azotaemia (creatinine > 140 μmol/L), LT4 replacement was advised. Levothyroxine could also be started in non-azotaemic cats with appropriate clinical signs (e.g., lethargy, weight gain, poor hair coat quality), but only if TT4 was below RR (TT4 < 12.9 nmol/L). Levothyroxine treatment was started at a dose of 100 µg/cat once daily (ideally given in the evening) and the dose was increased by 50 µg every four weeks until TT4 concentration of 32.2–45 nmol/L (measured 12 h post pill) was achieved [11,15].

### 2.6. Statistical Analysis

Statistical analysis was performed using commercially available statistical software (SPSS version 26; IBM Statistics Germany GmbH, Schorndorf, Germany). Data were tested for normality using Shapiro–Wilk test where appropriate. All numerical data were presented as median (range), proportions are presented as percentages (e.g., percentage of cats with eu-/hypo-/hyperthyroidism or percentage of cats with azotaemia at different timepoints; percentage of cats with very poor to very good QoL using the 5-point scale from the HyperthyroidismQoL-cat tool). Health-related quality-of-life was compared between groups at study enrolment by Mann–Whitney U tests. The overall effect of treatment group (ATD- vs. RAIT-group), timepoint (enrolment, month-1, month-3, month-6) and thyroid status (hyper-, eu-, hypothyroid [including cats under LT4 supplementation]) on HRQoL was assessed by mixed-effects modelling. To meet the assumptions of the model, HRQoL was transformed using log(HRQoL). Fisher’s least significant difference tests were used for post hoc comparisons when factors reported significant effect on outcome variable. Mann–Whitney U test was used to compare HRQoL scores of all study cats (irrespective of treatment modality) at month-6 with a non-hyperthyroid comparison group from a previously published study, using the same HRQoL tool [3], to assess whether HRQoL normalises following treatment or remains impaired as previously reported in hyperthyroid humans [7,8]. Changes in QoL scores per domain (“owner related”, “gastrointestinal, dietary, urination”, “appearance”, and “activity and behaviour”) between “enrolment” and “month-6” within the ATD- and RAIT-group were assessed using the Wilcoxon signed rank test. Fisher Exact Probability Test was used to compare the proportion of cats with improved owner-reported QoL (using the 5-point scale from very poor to very good from the HyperthyroidismQoL-cat tool) and cats with unchanged/poorer owner-reported QoL (grouped together for the purpose of statistical analysis) between the RAIT- and ATD-group at the cats’ last assessments. Fisher Exact Probability Test was also used to analyse differences in the proportions of cats that received treatment with ATD prior to study enrolment. Significance was set at *p* < 0.05.

## 3. Results

### 3.1. Study Cohort

A total of 40 cats were initially enrolled onto the study; 23 (57.5%) were in the RAIT-group and 17 (42.5%) in the ATD-group. Two cats of the ATD-group were excluded (one cat died before the second questionnaire was completed, and the owner of the other cat did not complete the second questionnaire despite three reminder emails), leaving 15 cats in the ATD-group. No cats were excluded from the RAIT-group. Of the 38 cats remaining in the study, 28/38 (74%) completed the study, with 19/28 (68%) belonging to the RAIT-group and 9/28 (32%) to the ADT-group. Of the ten cats that did not complete the study, five cats died during the six months study period (two before month-3 and three before month-6), and four owners elected to discontinue their participation in the study (one before month-3 and three before month-6). Furthermore, one cat in the ATD-group received RAIT before completing the month-3 questionnaire (i.e., only enrolment and month-1 data of this cat were included in the analysis). None of the five deceased cats had a cause of death that could be directly associated with hyperthyroidism or its treatment. Reported causes of death were development of facial squamous cell carcinoma, hepatocellular carcinoma, mediastinal thymoma, severe epilepsy and one cat died at night for no apparent reason; however, the owner did not want a post-mortem pathological examination

Of the 38 study cats, 21/38 (55%) cats were female-neutered, 15/38 (40%) cats were male-castrated and 2/38 (5%) were female-intact, there was no difference in the sex distribution between the RAIT and ATD groups (*p* = 0.80). The median age was 12 years (range 7.5–16 years) in the RAIT-group and 14 years (range 10–20 years) in the ATD-group.

Twelve (12/23, 52%) cats of the RAIT-group were pretreated with ATD for less than one month (2/12, 17%), one to three months (7/12, 58%) or three to six months (3/12, 25%) prior to RAIT. In the remaining 11/23 (48%) cats, ATDs were either given for a few days and discontinued because of adverse effects (8/11, 73%) or the cats were not given any medications at all because owners were unable (1/11, 9%) or did not want to (2/11, 18%) give tablets.

In the ATD-group, all cats were treated with some form of ATD prior to enrolment; 6/15 (40%) cats were treated with ATD for less than one month, 5/15 (33%) for one to three months, and 3/15 (20%) for three to six months. Fourteen (14/15, 93%) cats were treated with antithyroid tablets and 1/15 (7%) with a liquid formulation. For those cats that received some form of treatment prior to enrolment on the study, there was no significant difference in the distribution of treatment length prior to enrolment between ATD- and RAIT-group (*p* = 0.36).

A total of 10 of the 23 (43%) cats in the RAIT-group and 11/15 (73%) cats in the ATD-group had a comorbidity at enrolment (disease other than hyperthyroidism) (Table 1).

Scintigraphy in the RAIT cats revealed a unilateral adenoma in 15 (65%) cats, bilateral adenoma in 7 (30%) cats and carcinoma in 1 (5%) cat. Treatment outcome is described in Section 3.3.

Out of the 38 participants, one owner used the English version of the questionnaire, the remainder answered in German.

### 3.2. Health-Related Quality-of-Life

The HRQoL scores of all cats and of the RAIT-group and ATD-group cats at the four study timepoints are shown in Table 2.

**Figure 1:**
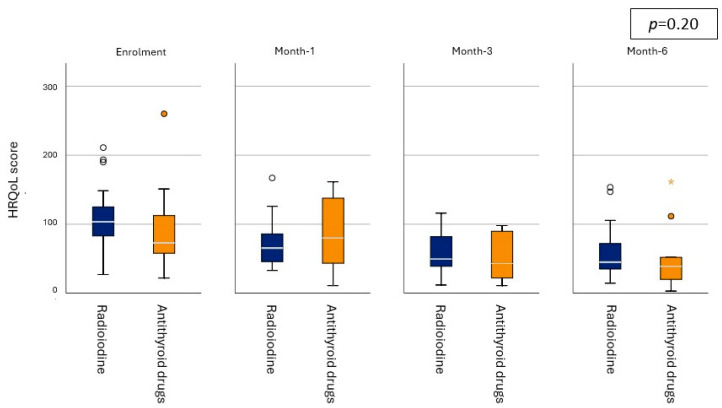
Box whisker plots depicting the health-related quality-of-life (HRQoL) score of cats in the radioiodine treatment group (blue) and the antithyroid drug group (orange) over the course of 6 months at the four study timepoints. The bold white line within the box shows the median value, boxes represent the interquartile range (IQR). The whiskers denote the range extending out to 1.5 × IQR, and data points that are >1.5 × IQR are shown as circles with the asterisk (*) representing an extreme value (more than three times the height of the boxes). Mixed effects modelling revealed no significant effect of treatment group.

**Figure 2:**
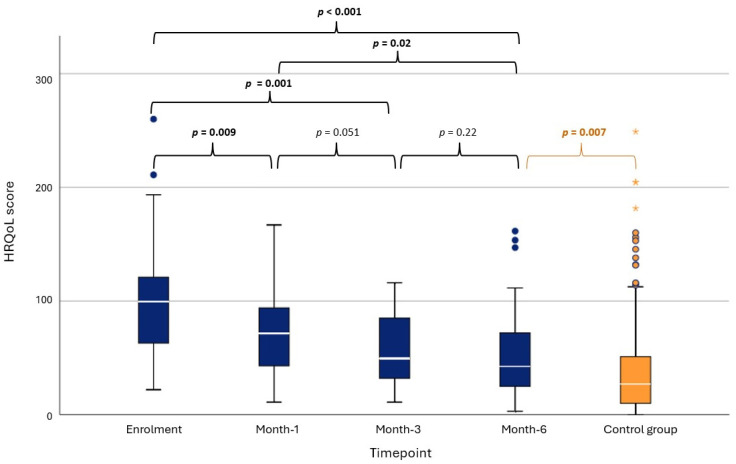
Box whisker plots depicting health-related quality-of-life (HRQoL) score of all study cats receiving either radioiodine treatment or antithyroid drug therapy (n = 38) over the course of 6 months at the four study timepoints (blue boxes) as well as HRQoL scores of a control group (orange box) without hyperthyroidism (n = 322), established in a previous study [3]. The bold white line within the box shows the median value, boxes represent the interquartile range (IQR). The whiskers denote the range extending out to 1.5 × IQR, and data points that were >1.5 × IQR are shown as circles with the asterisk (*) representing an extreme value (more than three times the height of the boxes). Mixed effects modelling revealed significant effect of study timepoint on the changes in HRQoL. Mann–Whitney U Test revealed a significant difference in HRQoL between all study cats at month-6 and a non-hyperthyroid control group. *p*-values are provided above the horizontal brackets, with significant *p*-values in bold and *p*-value calculated using Mann–Whitney U Test in orange font.

There was no difference in HRQoL score between the treatment groups upon enrolment (*p* = 0.22, see Table 2 for the scores). Although the changes in median HRQoL scores within the groups (Table 2, Figure 1) suggest a possible faster improvement in median HRQoL scores within the RAIT-group (within 1 month) when compared to the ATD-group (improvement within 3 months); mixed effects modelling revealed that there was no effect of the treatment group on the log(HRQoL) scores (*p* = 0.20). However, timepoint had a significant effect on the log(HRQoL) score (*p* < 0.001), with log(HRQoL) being higher (indicating poorer QoL) at enrolment when compared to all other timepoints (all *p* < 0.05, see Figure 2 for the exact *p*-values). Log(HRQoL) score was also significantly higher at month-1 when compared to month-6, but there was no difference between month-1 and month-3 and between month-3 and month-6 (Figure 2).

Comparing the HRQoL score at timepoint “month-6” to a previously established control population of 322 cats without hyperthyroidism (Supplementary material) that completed the HyperthyroidismQoL-cat tool as a part of a previous validation study between November 2021 and January 2022 [3], the HRQoL score was still significantly higher in the cats suffering from hyperthyroidism (study cats at month-6: median 42.5 (range 3–161.5); control population: median 27; range, 0–249, *p* = 0.007).

**Figure 3:**
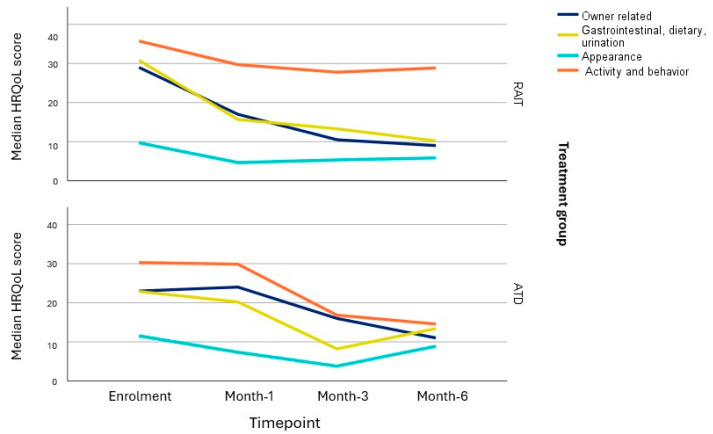
Changes in the health-related quality-of-life (HRQoL) scores in of each of the 4 domains of the HyperthyroidismQoL-cat questionnaire over a 6-month period in the radioiodine treatment group (RAIT) and the antithyroid drug group (ATD). Each domain is represented by a different colour. The lines connect median HRQoL scores of the 4 domains.

Changes in the HRQoL score per each of the four domains (“owner related”, “gastrointestinal, dietary, urination”, “appearance”, and “activity and behaviour”) are visualised in Figure 3. In the RAIT-group, significant improvement (i.e., lower HRQoL scores) from enrolment to month-6 was reached in the domains “owner related” (*p* = 0.02) and “gastrointestinal, dietary, urination” (*p* = 0.002) but not in the domains “appearance” (*p* = 0.09) and “activity and behaviour” (*p* = 0.5). In the ATD-group, only the domain “owner related” reached significant improvement (*p* = 0.02) at month-6 (“gastrointestinal, dietary, urination” *p* = 0.31, “appearance” *p* = 1.0, and “activity and behaviour” *p* = 0.14).

**Figure 4:**
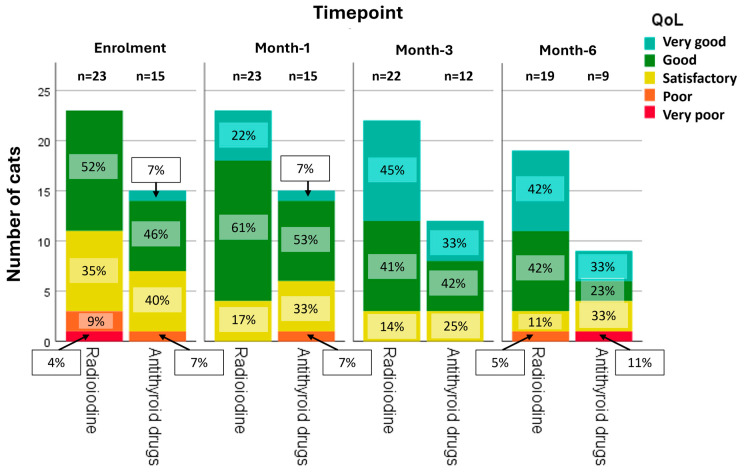
Bar chart depicting the owners’ rating of their cat’s overall quality-of-life (QoL) on a 5-point scale from very poor to very good at all four timepoints (enrolment, month-1, month-3, month-6) for both treatment groups (radioiodine and antithyroid drug group). Number (n) of cats with available QoL assessment and the percentage of cats with the respective rating (from very poor to very good) is provided at each timepoint within each group.

Besides the calculation of the total HRQoL score, the HyperthyroidismQoL-cat tool offers the option to assess the overall QoL on a 5-point scale (from very good to very poor). Owners’ assessment of the overall QoL of their cats using this 5-point scale upon enrolment and at all subsequent timepoints is depicted in Figure 4. In total, 19/38 (50%) owners reported a better QoL of their cat (meaning an improvement of at least 1-point on the scale, e.g., from very poor to poor) at their last completed questionnaire when compared to enrolment, 15/38 (39.5%) reported an equal/unchanged QoL, and 4/38 (10.5%) reported a worse QoL at their cat’s last assessment. When looking at the groups separately, in the RAIT-group, 13/23 (57%) owners reported a better QoL, 9/23 (39%) reported the same QoL, and 1/23 (4%) reported a worse QoL, while in the ATD-group, 6/15 (40%) owner reported a better QoL, 6/15 (40%) had an unchanged QoL, and 3/15 (20%) a worse QoL at their last assessment. When the proportion of cats with improved HRQoL and cats with unchanged/poorer HRQoL (grouped together for the purpose of statistical analysis) at the cat’s last assessment was compared between the RAIT- and ATD-group, there was no significant difference (*p* = 0.36).

### 3.3. Thyroid Status and Its Effect on HRQoL

**Figure 5:**
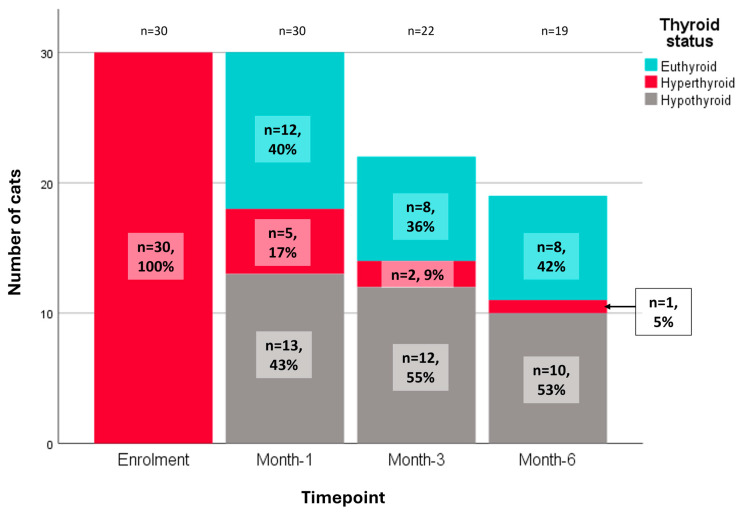
Bar chart depicting the number of cats with known thyroid status (hypo-, eu-, hyperthyroid) at all four study timepoints (enrolment, month-1, month-3, month-6) from both treatment groups. n: number of cats with available data at the respective timepoint and number of cats within the respective thyroid status category.

Thyroid status could be assessed in 30 cats (RAIT-group: 23; ATD-group: 7) at enrolment, in 30 cats (RAIT-group: 23; ATD-group: 7) at month-1, in 22 cats (RAIT-group: 22; ATD-group: 0) at month-3, and in 19 cats (RAIT-group: 17; ATD-group: 2) at month-6. Thyroid status at enrolment and the follow-up timepoints is presented in Figure 5.

Upon enrolment, all 30 cats with available blood work had hyperthyroidism. In the ATD-group, out of the available nine TT4 results over the 6 months follow-up period, two cats were hyperthyroid at month-1 and all remaining TT4 results were within euthyroid range. In the RAIT-group, TT4 was within or below RR in 20/23 (87%) at month-1 and normalised in one additional cat at month-3 (i.e., hyperthyroidism resolved in 21/23 [91%] RAI-treated cats). In the other 2/23 (9%) cats that were hyperthyroid at month-1, one left the study at month-3 (still hyperthyroid) and the other one completed the study but was still hyperthyroid at month-6. Of the 17/23 (74%) RAIT-group cats that completed the 6-month study period, 6/17 (35%) were euthyroid, 10/17 (59%) were hypothyroid (5/10 receiving LT4 supplementation) and 1/17 (6%) (mentioned above) was hyperthyroid at the final month-6 re-examination. Three RAIT-group cats (3/23; 13%) left the study after month-3, of which 1/3 was euthyroid, 1/3 was hypothyroid (not receiving LT4 supplementation) and 1/3 was hyperthyroid (mentioned above). A single (1/23; 4%) RAIT-group cat left the study after month-1, with test results indicating hypothyroidism. Due to the low number of cats with known thyroid status during the later timepoints in the study, statistical analysis on distribution of thyroid status across treatment groups was not conducted.

**Figure 6:**
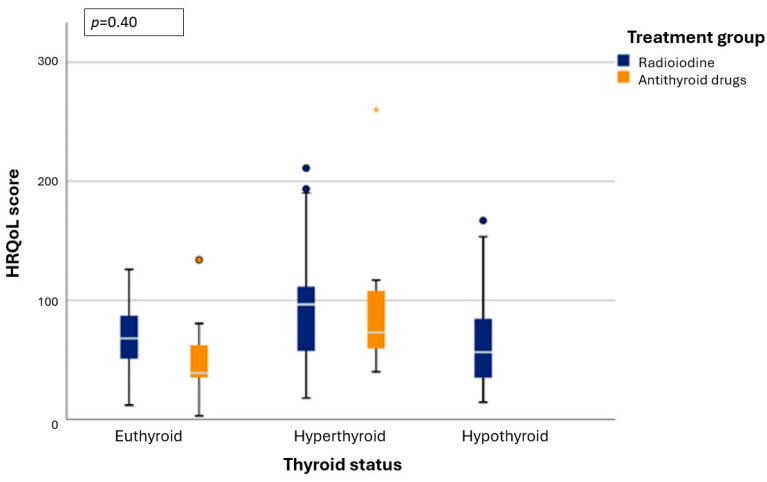
Box whisker plots depicting the health-related quality-of-life (HRQoL) score of cats in the radioiodine treatment group (blue) and the antithyroid drug group (orange) across thyroid status categories (hyper-/eu-/hypothyroid) including all HRQoL results of all cats with known thyroid status over all four timepoints presented by treatment group. The bold white line within the box shows the median value, boxes represent the interquartile range (IQR). The whiskers denote the range extending out to 1.5 × IQR, and data points that were >1.5 × IQR are shown as circles with the asterisk (*) representing an extreme value (more than three times the height of the boxes). Mixed effects modelling revealed no significant effect of thyroid status on changes in HRQoL.

Distribution of HRQoL scores across thyroid status categories (hyper-/eu-/hypothyroid [including cats under LT4 supplementation]) including all HRQoL results of all study cats (both RAIT- and ATD-group) with known thyroid status across all four study timepoints is in Figure 6. Mixed effect modelling revealed that there was no significant effect of thyroid status on HRQoL (*p* = 0.40).

### 3.4. Azotaemia

Measurement of creatinine concentration was available for 30 cats (RAIT-group: 23; ATD-group: 7) at enrolment, in 29 cats (RAIT-group: 23; ATD-group: 6) at month-1, in 21 cats (RAIT-group: 21; ATD-group: 0) at month-3, and in 17 cats (RAIT-group: 16; ATD-group: 1) at month-6. The presence or absence of azotaemia at the respective study timepoints is summarised in Table 3. Of the 97 available creatinine results, 21/97 (22%) indicated the presence of azotaemia. Azotaemia was mild (IRIS stage 2, 140–250 μmol/L) [9] and not considered clinically relevant in all but 1/21 occasions (5%). Therefore, the effect of clinically significant azotaemia (IRIS stage 3 or 4) on HRQoL could not be assessed.

## 4. Discussion

The main aim of this study was to assess whether there is an effect of treatment modality (RAIT vs. ATD) on changes in HRQoL of hyperthyroid cats over a 6-month period. Secondary aim was to assess if thyroid status (hyper-/eu-/hypothyroid) and clinically relevant azotaemia have an effect on changes in HRQoL and whether the HRQoL remains impaired six months after treatment compared to a non-hyperthyroid control group. The study revealed that HRQoL scores improved in both treatment groups (RAIT vs. ATD) over the 6-month period, with a significant improvement seen already after one month, but the treatment group (and therefore treatment modality) did not influence HRQoL. Additionally, thyroid status did not have a significant effect on changes in the HRQoL. Because only one cat had clinically relevant azotaemia (IRIS Grade 3 or 4 [9]), no statistical evaluation of the influence of this factor on HRQoL was possible. At the 6-month’s follow-up, HRQoL of our study cats was still lower when compared to non-hyperthyroid controls.

Our findings indicating rapid improvement of HRQoL following treatment of hyperthyroidism are in agreement with a recent questionnaire-based study from Belgium looking at owners’ perceptions regarding RAIT, in which owners noted an improvement of clinical signs within one month (46%) or within 1–6 months (34%) of RAIT, alongside significant increase in QoL-score (rated on a scale from 1 to 10) [16]. Similar improvement in QoL rated on a 10-point scale was reported in another survey assessing owners’ experiences with RAIT in United Kingdom [17]. In contrast to our study, a validated QoL-tool was not used previously [16,17] and QoL was evaluated retrospectively, which in some cats, especially in the Belgian study, meant that several years have elapsed between RAIT and completion of the survey, potentially causing recall bias [18]. On the other hand, six months following RAIT or ATD-treatment start, the HRQoL of our study cats was still lower when compared to non-hyperthyroid control group (from a previous study) [3]. This is similar to findings reported in people with hyperthyroidism. Although improvement of QoL scores is generally seen in people with hyperthyroidism after reaching euthyroidism or after one year of therapy, HRQoL remains worse for prolonged periods of time (even years after/under treatment) when compared to a non-hyperthyroid control group [6,7,8], regardless of the treatment modality [6,8,19]. This prolonged negative influence of hyperthyroidism on the HRQoL in people might be related to the mental aspects of the disease, including social limitations [6]. In veterinary medicine, the concept of mental health is much more difficult to capture and quantify, even with validated HRQoL tools. However, it can be assumed that the influence in animals is less pronounced, as social limitations probably only have a negligible influence on HRQoL. Still, several reasons for lower HRQoL in our study cats following six months of treatment when compared to non-hyperthyroid controls should be considered, including the need for ongoing monitoring, lack of hyperthyroidism control in some cats (two cats of the RAIT-group remained hyperthyroid), side effects of ATD, need for hypothyroidism treatment or treatment of chronic kidney disease. It must be further noted that the median age of cats in our study (RAIT cats 12 years, ATD cats 14 years) was higher than that of the used control group (5 years), which may have influenced the observed differences in HRQoL Age-related factors and the presence or progression of concurrent diseases could have contributed to the lower scores in treated hyperthyroid cats.

Similarly to human medicine [5,6,7], using mixed effects modelling, our study also revealed no effect of treatment modality (RAIT vs. ATD) on changes in HRQoL following therapy. Although, using the 5-point scale included in the HyperthyroidismQoL-cat tool, there was a numerical difference in the proportion of cats with improved QoL at their study endpoint (57% in the RAIT-group vs. 40% in the ATD-group), which also was not significant. These findings differ from results of a survey conducted in Switzerland, where owners rated their cats’ QoL significantly better after RAIT, compared to owners of cats treated with ATD [20]. However as far as can be seen from that conference abstract, no independently validated QoL tool was used for this assessment [20], and therefore the results of our and the Swiss study might not be directly comparable.

Our results indicating a lack of effect of treatment modality on HRQoL, however, are in contrary to our expectation of the RAI-treated cats to experience better HRQoL in comparison to cats treated with ATD. We expected that owners of the RAIT-group would rate at least the “owner”-related questions (e.g., daily life restrictions, emotional stress of the owner, financial burden, need for daily medication administration, possible side effects of the treatment, and necessary follow up) in the HRQoL questionnaire better, as no regular treatment or ongoing financial and emotional burden is expected after RAIT. However, in this study, both RAI- and ATD-treated cats had a significant improvement in the domain “owner related” at the end of the 6-month study period. Possibly, RAIT might have had a negative influence on owner-related questions due to concerns about hospitalisation length (due to radiation protection legislation), development of comorbid diseases and side effects, which were the main concerns of owners of RAI-treated cats in the previously mentioned study from United Kingdom [17]. These concerns might have negatively affected the owners’ perception of their cats’ QoL following RAIT and explain why the anticipated difference between the RAIT- and ATD-groups was not identified. Another possible reason for the lack of effect of treatment modality on HRQoL was the small sample size, with only 38 cats included in the analysis. Furthermore, a number of cats in our study received treatment prior to enrolment, which might have had some impact on their QoL. It can be expected that some of the pre-treated cats had fewer clinical signs at enrolment than before starting therapy, as clinical signs usually improve once a cat has a TT4 level within the RR for 2 to 6 weeks, and normal TT4 levels are mostly reached within 2 to 4 weeks of initiating therapy with ATD [10,21,22]. However, the frequency and duration of pre-treatment were comparable between treatment groups, and therefore the effect of pre-treatment on the results of our study is likely minimal, if any.

Influence of thyroid status on HRQoL was also assessed in this study, but no significant effect could be shown. This could be because only a very small proportion of cats remained hyperthyroid during therapy with ATD or after RAIT and cats with hypothyroidism were supplemented with thyroid hormones, therefore mitigating most factors that might have worsened HRQoL scores. Furthermore, the number of cats with hypothyroidism might have been underestimated, especially in the ATD-group, as TSH measurement was only available in RAI-treated cats. However, in elderly humans, it has been shown that subclinical and overt hypothyroidism does not lead to a reduction in HRQoL [23,24], which seems in agreement with our results. Although it was surprising that mixed effect modelling did not identify worse HRQoL in hyperthyroidism (when compared to eu- and possibly also hypothyroidism), the most likely reason for the lack of significance was the small sample size and the fact that thyroid status was mainly known in the RAIT-group cats, with very few results available for the ATD-group cats. It should be noted that the rate of hypothyroidism observed in our study following RAIT was relatively high (59%), but falls within the range reported in previous studies. For instance, a study using a fixed 4 mCi dose reported a hypothyroidism rate of 64% (25/39 cats) [13], and another study using an individualised dosing protocol found a rate of 53% (29/55 cats) [25]. In both studies, thyroid function was assessed at six months [13] or between six- and nine-months post-RAIT [25]. It must be further noted that, TSH was measured for the RAIT cats. Cats with a total thyroxine (T4) concentration in the lower reference range combined with an elevated TSH were classified as hypothyroid, including those with subclinical hypothyroidism. This broader diagnostic criterion likely contributes to the higher prevalence of hypothyroidism observed in our study compared to previous studies that relied solely on T4 concentrations below the reference range for diagnosis, without incorporating TSH values.

The possible influence of clinically relevant azotaemia on HRQoL could not be analysed in our study, because at all but one occasion the azotaemia was mild (IRIS grade 2) [9]. With mild azotaemia, significant influence on HRQoL is unlikely, as only mild clinical signs are expected with this level of azotaemia. Additionally, some cats with azotaemia were also hypothyroid at those timepoints, and afterwards started on LT4 supplementation, again reducing the expected clinical signs.

The main limitation of this study is the small sample size, combined with the laboratory reassessment examinations which could not be completely enforced at the standardised timepoints, as the medical care was continued by the family veterinarian in the ATD-group, and owner compliance could not be enforced, which resulted in missing data. Due to this missing data, some cats could not be assigned according to thyroid status at the follow-up timepoints. This made it particularly difficult to assess the influence of thyroid status on HRQoL. As mentioned above, this also resulted in a non-uniform classification of hypothyroidism in the RAIT and ATD groups. This limitation could not be eliminated in our study, as blood samples were not taken without medical indication which was decided by the primary veterinarian. Additional limitations were that the group sizes were not identical and there was no randomised allocation to treatment groups, which was based on the owners’ treatment decision. Randomisation was not possible due to the clinical nature of the study and significant difference in treatment modalities, including costs and expected side effects/risks and associated temporary restrictions, such as radiation protection requirements. This is a frequent encountered limitation in clinical trials in veterinary medicine when treatment options strongly vary in invasiveness and cost and owner decision is included in the decision [26,27,28]. This means that owner characteristics, which might have influenced the choice of treatment, might also have had an impact on the HRQoL score. However, the influence on the course of the HRQoL score should be minimal, as the HRQoL questionnaire was always completed by the same owner. While RAIT was standardised, this was not possible for the ATD group, as some of the cats were already under treatment by their primary veterinarian at the time of enrolment. Furthermore, a high number of cats in both treatment groups (RAIT 43%, ATD 73%) were suffering from a comorbidity other than hyperthyroidism. In our previous study that developed and validated the HyperthyroidismQoL-cat questionnaire, we have demonstrated that comorbidities can have an influence on the HRQoL score [3]. However, the specific comorbidities (not only the organ system affected), their clinical manifestation, and their progression during the six months period were not recorded and the influence on the HRQoL score could therefore not be determined, posing a considerable limitation to this study, in particular because the number of cats with a comorbidity was different between the RAIT group and the ATD group (RAIT 43%, ATD 73%). Cats with comorbidities were included in the study for a number of primary reasons. First, due to the advanced age typically associated with hyperthyroid cats, comorbid conditions are prevalent, making the recruitment of a sufficiently large cohort of comorbidity-free individuals unfeasible. Second, the original study that developed and validated the questionnaire also included cats with comorbidities in both the hyperthyroid and control groups, thereby ensuring methodological consistency and allowing for valid comparisons. Furthermore, the number of cats with comorbidities remained constant throughout the study period. The median HRQoL did not differ significantly between the RAIT and ATD groups at baseline despite the different number of comorbidities. As the study was designed to assess changes within treatment groups over time, and the distribution of comorbidities within each group remained stable across timepoints, we consider the potential impact of comorbid conditions on the study outcomes to be minimal and acceptable. Comorbidity is acknowledged as a limiting factor in several human studies comparing treatment outcomes in terms of HRQoL; notably, in one study, repeating analyses with adjustment for comorbidities did not alter the results, thereby supporting our decision to include cats with comorbidities [7]. Furthermore, many studies evaluating the QoL of specific diseases and their treatment have not accounted for concurrent conditions, potentially underestimating their impact on HRQoL assessments [29], or only excluded certain severe comorbidities [4]. In veterinary medicine, comorbidities have also been reported as a factor influencing HRQoL to a certain extent. The possible effect on measured HRQoL may differ depending on the number, severity, and progression of the conditions involved [30]. These findings underscore the importance of considering comorbidities when assessing HRQoL in both veterinary and human populations. While comorbidities can complicate treatment outcomes and quality of life assessments, they do not necessarily diminish the effectiveness of the analysis. Therefore, including patients or animals with comorbidities in studies is crucial for obtaining a comprehensive understanding of treatment impacts on HRQoL. An additional limitation is that five cats died during the study period. Even if their respective causes of death were not related to hyperthyroidism or its therapy, a negative influence on the HRQoL score of the disease leading to death must be assumed, posing another limitation to this study.

## 5. Conclusions

This study was able to show that the HRQoL of hyperthyroid cats, measured using the validated HyperthyroidismQoL-cat questionnaire, improves significantly within six months both under ATD therapy and following RAIT, with improvement in HRQoL being detected as early as one month following enrolment. Neither treatment modality (RAIT vs. ATD) nor thyroid status had an effect on HRQoL in this study. However, further studies are warranted to fully explore these variables due to the small number of included cats, the lack of randomisation and heterogeneity of subjects and treatment; therefore, conclusion have to be considered preliminary.

## Figures and Tables

**Table 1:** Absolute and relative number or cats with comorbidity and affected organ system/disease complex for cats in the antithyroid drug (ATD) group and radioiodine treatment (RAIT) group.

Treatment Group (Number of Included Cats)	ATD (n = 15) n (%)	RAIT (n = 23) n (%)
Comorbidity		
No comorbidity reported	4 (27%)	13 (57%)
Any comorbidity	11 (73%)	10 (43%)
Chronic kidney disease	2 (13%)	0 (0%)
Musculoskeletal disorder	3 (20%)	1 (4%)
Skin disease	0 (0%)	2 (9%)
Gastrointestinal disease	3 (20%)	0 (0%)
Dental disease	7 (47%)	2 (9%)
Respiratory tract disease	0 (0%)	2 (9%)
FIV or FeLV	1 (7%)	0 (0%)
Cardiac disease	1 (7%)	5 (22%)
Not further classified	0 (0%)	2 (9%)

**Table 2:** Health-related quality-of-life score of all studied cats, cats in the radioiodine treatment (RAIT) group (n = 23) and antithyroid drug (ATD) group (n = 15) at the four study timepoints (enrolment, month-1, month-3 and month-6).

Timepoint	Enrolment n Median (Range)	Month-1 n Median (Range)	Month-3 n Median (Range)	Month-6 n Median (Range)
Subgroup				
All cats	n = 38 99.6 (22–260)	n = 38 71.5 (11–167)	n = 34 49.5 (11–116)	n = 28 42.5 (3–161.5)
RAIT	n = 23 103.5 (27–211)	n = 23 65.5 (33–167)	n = 22 49.5 (12–116)	n = 19 45 (14.5–153.5)
ATD	n = 15 73 (22–260)	n = 15 80 (11–161)	n = 12 43.0 (11–98)	n = 9 39.0 (3–161.5)

Abbreviations: n = number of cats.

**Table 3:** Number and percentage of cats per group (RAIT group [radioiodine-treated cats), ATD group [cats treated with antithyroid drugs]) with and without azotaemia at each study timepoint.

Timepoint	Enrolment		Month-1		Month-3		Month-6	
Renal Status	Azotaemic	Non-Azotaemic	Azotaemic	Non-Azotaemic	Azotaemic	Non-Azotaemic	Azotaemic	Non-Azotaemic
Treatment Group								
RAIT group	0 (0%)	23 (100%)	5 (22%)	18 (78%)	7 (33%)	14 (67%)	4 (25%)	12 (75%)
ATD group	1 (14%)	6 (86%)	3 (50%)	3 (50%)	0 (0%)	0 (0%)	1 (100%)	0 (0%)
Total	1 (3%)	29 (97%)	8 (28%)	21 (72%)	7 (33%)	14 (67%)	5 (27%)	13 (72%)

## Data Availability

All the results of the study are presented in the manuscript and in the Supplementary Materials.

## Supplementary Materials

The following supporting information can be downloaded at: https://www.mdpi.com/article/10.3390/vetsci12060572/s1. Characterisation of the control group used and study results.

## Abbreviations

The following abbreviations are used in this manuscript:

ATD	Antithyroid drug therapy
CKD	Chronic kidney disease
HRQoL	Health-related quality-of-life
IRIS	International Renal Interest Society
IQR	Interquartile range
LT4	Levothyroxine
QoL	Quality-of-life
RAIT	Radioiodine treatment
TSH	Thyroid stimulating hormone
TT4	Total thyroxine
