# Supplementary material for: Comparison of the Changes in Quality-of-Life of Cats with Hyperthyroidism Treated with Radioiodine or Antithyroid Drugs—A Preliminary Study †
Source: Vet Sci. 2025 Jun 11;12(6):572. doi: 10.3390/vetsci12060572 (PMC12197586; doi:10.3390/vetsci12060572)
Supplement: Supplementary file 1 [file vetsci-12-00572-s001.zip › Supplementary1.pdf]

Supplementary file 1

Characterisation of the control group used (based on Blunschi et al. Development and validation of a questionnaire to assess health-related quality-of-life in cats with hyperthyroidism. *J Vet Intern Med*. 2024)

The control group consisted of 322 non-hyperthyroid cats from a previous validation study. The median age of the cats was 5 years (range 0 – 21, IQR 2 – 9). The median HRQoL score was 27 (range 0 – 249, IQR 10 – 51). The following table shows the comorbidities of the non-hyperthyroid control group.

Comorbidity	Control-group<br><br>n (%)
Cats with known comorbidity status	295
No comorbidity	163 (55.3%)
Any comorbidity	132 (44.8%)
Dental disease	25 (8.5%)
Chronic kidney disease	19 (6.4%)
Gastrointestinal disease	20 (6.8%)
Musculoskeletal disorder	20 (6.8%)
Cardiac disease	15 (5.1%)
Respiratory tract disease	12 (4.1%)
Urinary tract disease	14 (4.8%)
Diabetes mellitus	9 (3.1%)
Skin disease	6 (2.0%)
FIV or FeLV	2 (.7%)
Not further classified	22 (7.5%)
